# Synthesis, Structural Characterization, and Cytotoxic Activity of Novel Paramagnetic Platinum Hematoporphyrin IX Complexes: Potent Antitumor Agents

**DOI:** 10.1155/2007/67376

**Published:** 2007-08-08

**Authors:** G. Gencheva, D. Tsekova, G. Gochev, G. Momekov, G. Tyuliev, V. Skumryev, M. Karaivanova, P. R. Bontchev

**Affiliations:** ^1^Department of Analytical Chemistry, Faculty of Chemistry, St. Kliment Ohridsky University of Sofia, 1 J. Bourchier Boulevard, 1164 Sofia, Bulgaria; ^2^Department of Pharmacology and Toxicology, Faculty of Pharmacy, Medical University of Sofia, 2 Dunav, 1000 Sofia, Bulgaria; ^3^Institute of General and Inorganic Chemistry, Bulgarian Academy of Sciences, Acad. G. Bonchev Street Bl. 11, 1113 Sofia, Bulgaria; ^4^Departamento de Fisica, Institució Catalana de Recerca i Estudis Avancats (ICREA), Universitat Autònoma de Barcelona, Bellaterra, 08193 Bellaterra, Spain

## Abstract

Three novel stable Pt(III) complexes with distorted octahedral structure and ${\left({\text{d}}_{{\text{z}}^{2}}\right)}^{1}$ ground state have been obtained in the course of Pt(II)-hematoporphyrin IX ((7,12-bis(1-hydroxyethyl)-3,8,13,17-tetramethyl-21H-23H-porphyn-2,18-dipropionic acid), Hp) interaction in alkaline aqueous medium and aerobic conditions. A redox interaction also takes place together with the complexation process leading to the formation of Pt(III) species and organic radicals. The processes in the reaction system and the structure of the complexes formed *cis*-[Pt(III)${\left({\text{NH}}_{3}\right)}_{2}$(${\text{Hp}}_{-3\text{H}}$)${\left({\text{H}}_{2}\text{O}\right)}_{2}$]$\cdot {\text{H}}_{2}\text{O}$ **1**, [Pt(III)(${\text{Hp}}_{-3\text{H}}$)${\left({\text{H}}_{2}\text{O}\right)}_{2}$]$\cdot {\text{H}}_{2}\text{O}$ **2**, and [Pt((O,O)${\text{Hp}}_{-2\text{H}}$)Cl${\left({\text{H}}_{2}\text{O}\right)}_{3}$] **3**, were studied by UV-Vis, IR, EPR and XPS spectra, thermal (TGS, DSC), potentiometric and magnetic methods. The newly synthesized complexes show promising cytotoxic activity comparable with that of *cis*-platin in in vitro tests against a panel of human leukemia cell lines. The observed cytotoxicity of the complex **2** against SKW-3 cells (KE-37 derivative) is due to induction of cell death through apoptosis.

## 1. INTRODUCTION

Since the discovery of the cytotoxic activity of *cis*-platin (*cis*-diamminedichloroplatinum(II)) [1], the efforts have been directed towards elucidation of the molecular mechanism of its action [2–4] and synthesis of new platinum compounds with improved antitumor activity [5] and side effects. Till now, a large library of platinum complexes has been synthesized and their antitumour activity has been examined but only *cis*-platin, carboplatin, and oxaliplatin have received a worldwide approval and achieved routine clinical use. At present, the cytotoxicity of *cis*-platin and other platinum(II) complexes is thought to originate from their interaction with DNA as well as with non-DNA targets [2, 6–8], and subsequent induction of cell death through apoptosis and/or necrosis [9, 10]. Despite its wide application as an antitumor drug for treatment of different kinds of cancer, *cis*-platin has several inherent shortcomings as limited solubility and side toxic effects including nausea, nephrotoxicity, vomiting, and so forth [11]. In addition, many tumors display natural resistance to *cis*-platin and others develop resistance after the initial treatment [12]. Thus the application of the drug is restricted to a relatively narrow class of tumors and the efforts were focused on the design of new platinum compounds with broader spectrum of antitumor activity and improved pharmacological properties with respect to toxicity and resistance. Several complexes structurally related to *cis*-platin, such as oxaliplatin and carboplatin, nedaplatin (Japan), lobaplatin (China), heptaplatin (South Korea) [5, 13, 14], are currently in clinical trials and use, but till now they have not demonstrated significant advantages over *cis*-platin. It seems possible that the complex structural analogs of *cis*-platin designed on the basis of structure-activity relationships [15] could scarcely offer serious advantages over the existing drugs. Analyzing the current status of the platinum-based antitumor drugs [5], it can be concluded that the search for improved platinum antitumor agents continues [16–18] mainly in direction to modulate the DNA-binding mode and DNA damage by changing the model structure of the platinum drug used.

One of the strategies for a design of new platinum antitumor compounds is to combine platinum(II) moiety with proper carrier groups, and thus to target selectively the tumor cells. An example of this approach is the synthesis of porphyrin-platinum complexes. The porphyrin ligands provide preferable accumulation in neoplastic tissue [19–21], whereas platinum complexes such as *cis*-platin and carboplatin penetrate unselectively [3, 17]. Besides, the porphyrins and, in particular, hematoporphyrin derivatives (HpD) are known with their widespread application in the photodynamic therapy and diagnosis [22, 23]. During the last decades, a considerable interest has been paid to the synthesis and characterization of Pt(II) complexes with hematoporphyrin IX and hematoporphyrin-type ligands [21, 24–28]. The porphyrin ligand could be coordinated to Pt(II) as a bidentate or tetradentate one by the pyrrole nitrogens [24, 25]. Recently, the preparation and structural characterization of a large number of platinum-porphyrin conjugates have been discussed [21, 26–29]. In these compounds, platinum(II) fragment is attached to the propionic acid side chains out of the porphyrin macrocycle. The cytostatic activity of these platinum(II) porphyrin conjugates towards different kinds of mammary and bladder cancer cell lines has been tested in dark and after irradiation. A high activity was reported, especially for the water-soluble conjugates [27, 28].

Other factor of great importance for the enhancement of the antitumor activity and circumvention of the drug resistance is the oxidation state of platinum in the antitumor drug. The higher oxidation platinum complexes possess increased opportunity to be delivered to the cellular target as an active agent. It is well known that in physiological media, platinum(IV) anticancer agents are easily reduced to active platinum(II) products [5, 18]. An advantage of the Pt(IV) complexes is their lower reactivity which decreases the loss of the active drug and the incidence of the side reactions. In addition, the axial ligands in the octahedral platinum (IV) complexes could alter the lipophilicity and the redox potential of the complex, and thus improve the cellular uptake and control of the reduction rate.

In the present paper, we propose a new approach for design of cytotoxic agents with improved properties, namely to combine platinum in the unusual oxidation state +3 with a proper ligand system which could stabilize this oxidation state and serve as a specific carrier group. This approach is based on the idea for the intermediate behavior of Pt(III) complexes between the complexes of Pt(II) and Pt(IV) with respect to kinetic inertness and thermodynamic stability. Hematoporphyrin IX was used as a ligand system. As a further development of our studies on the synthesis of stable Pt(III) complexes [29–31] with proper ligand systems [32–34], we have investigated the Pt(II)-hematoporphyrin IX interaction in aqueous-alkaline medium and aerobic conditions on light. Three stable octahedral Pt(III)-hematoporphyin complexes have been synthesized and structurally characterized by spectroscopic and magnetic methods. The cytotoxic activity study showed that despite the violation of the empirical structure—activity relationship rules [15], the complexes show a promising cytotoxicity comparable with that of *cis*-platin. Although the high antitumor activity of mixed oxidation Pt(II)–Pt(III) polymeric complexes of “platinum blues” type was recognized long ago [35], this is the first report on the cytotoxic effect of stable monomer Pt(III) species.

## 2. MATERIALS AND METHODS

All reagents and solvents were of analytical grade and were obtained by commercial sources and used without further purification. A sample of 0.030 g (0.050 mmol) Hp was dissolved by stirring in 8 mL $5\times 10^{-2}$ M KOH (0.40 mmol) and a pH value of 11.0–11.5 was finally obtained. A *cis*-diammine(diaqua)platinum(II) hydroxide solution was prepared by stirring *cis*-platin (0.2 mmol, 0.060 g for the complex **1**; and 0.1 mmol, 0.030 g for the complex **2**) with 1.95 molar equivalents of AgNO_3_ in water (5 mL). The mixtures were kept in dark for 24 hours and the AgCl formed was then removed by a centrifuge. Aqueous solution of K_2_PtCl_4_ (1.10^−2^ M) was used for the synthesis. The pH values together with the UV-VIS and EPR spectra were monitored during the reaction course at regular intervals. The UV-VIS spectra were recorded after dilution of samples from the reaction mixtures with distilled water to $6.25\times 10^{-5}$ M ligand concentration. The EPR spectra of frozen samples (100–270 K) of the reaction systems were recorded without dilution.

### 2.1. Syntheses

#### 2.1.1. Preparation of cis-[Pt(NH_3_)_2_(Hp_−3H_)(H_2_O)_2_]·H_2_O (1)

Aqueous solution of diammine(diaqua)platinum(II) hydroxide (1.10^−2^ M, 0.2 mmol) was added in excess to a (0.05 mmol) solution of Hp (molar ratio Pt:Hp = 4) at permanent stirring. A solution of $5\times 10^{-2}$ M KOH was added immediately until pH 10.8–11.0 is obtained. During this period, the acidity of the reaction solution decreases further spontaneously, and at pH ∼ 8, the complex [Pt(NH_3_)_2_(Hp_−3H _)(H_2_O)_2_]·H_2_O **1** precipitates as a dark-violet powder. The powder was filtered, washed with water and alcohol, and dried over P_4_O_10_. Yield is 8.4 mg (19%). Water and ammonia were determined thermogravimetrically. The mass losses of 2.02%, 4.16%, and 3.67% in the temperature ranges 95–130°C, 130–260°C, and 260–295°C, respectively, correspond to 1H_2_O molecule, 2H_2_O molecules, and 2NH_3_ molecules per mol of the complex (theoretical contents 2.05%, 4.10%, and 3.88%, resp.). Formula C_34_H_47_N_6_O_9_Pt (878.8): *Anal.* Calc. C 46.46, H 5.39, N 9.56, Pt 22.19, found C 46.51, H 5.48, N 9.62, Pt 22.38%. IR (cm^−1^): 3303 ν(=NH); 3235, 3218, 3172, 3093 ν ^as,s^(NH_3_); 2964, 2924, 2855 ν ^as,s^(CH_3_, CH_2_); 1552, 1407 ν ^as,s^(COO^−^). UV-Vis (8.10^−5^ M in concentrate CH_3_COOH) λ_max_(log ε): 395 (4.87), 510 (3.86), 545 (3.92), 565 (3.90), 630sh (3.53).

#### 2.1.2. Preparation of [Pt(Hp_−3H_)(H_2_O)_2_]·H_2_O (2)

A procedure similar to that described for **1** was used for the preparation of the complex **2**, but the aqueous solution of diammine(diaqua)platinum(II) hydroxide (1.10^−2^ M, 0.1 mmol) was added in twofold excess (molar ratio Pt:Hp = 2) and a pH value of 10.3–10.5 was obtained. The complex with composition [Pt(Hp_−3H _)(H_2_O)_2_]·H_2_O **2** was isolated as a dark-violet precipitate by adding 3 mL 0.2M HNO_3_. The precipitate was filtered, washed with water and alcohol, and dried over P_4_O_10_. Yield is 34.6 mg (82%). The water content of the complex was found thermogravimetrically. The mass losses of 2.00% in the temperature range 100–130°C and 4.25% in the temperature range 130–250°C correspond to 1H_2_O molecule and 2H_2_O molecules (theoretical contents of 2.13% and 4.26%, resp.). Formula C_34_H_41_N_4_O_9_Pt (844.8): *Anal.* Calc. C 48.33, H 4.89, N 6.63, Pt 23.09, found C 48.26, H 4.79, N 6.70, Pt 23.15%. IR (cm^−1^): 2968, 2915, 2875, 2820sh ν ^as,s^(CH_3_, CH_2_); 1704 ν(−C=O); 1563, 1352, ν ^as,s^(COO^−^). UV-Vis (8.10^−5^ M in concentrate CH_3_COOH) λ_max_ (log ε): 400 (4.89), 520 (3.92), 560 (3.97), 635 sh (3.56); (8.10^−5^ M in DMF) λ_max_(log ε): 405 (4.88), 515 (3.94), 540 (3.94), 630 sh (3.49).

#### 2.1.3. Preparation of [Pt((O,O)Hp_−2H _)Cl(H_2_O)_3_] (3)

Aqueous solution of K_2_PtCl_4_ (0.0208 g, 0.05 mmol) and a solution of Hp (0.05 mmol) were mixed in equimolar ratio (Pt:Hp = 1) and the basicity of the reaction mixture was adjusted to pH = 11.5 by addition of $5\times 10^{-2}$ M KOH. The system was kept at ambient temperature for 2 weeks. A dark violet complex with composition [Pt((O,O)Hp_−2H _)Cl(H_2_O)_3_] was precipitated with 2 mL 0.2M HClO_4_. Yield is 40.1 mg (97%). The water content of the complex was found thermogravimetrically. The mass losses in the temperature ranges 100–161°C (4.09%) and 225–250°C (2.04%) correspond to 2H_2_O and 1H_2_O molecules (theoretical contents 4.09% and 2.04%, resp.). A mass loss of 4.14% observed in the temperature range 160–225°C corresponds to removal of Cl^−^ as HCl (theoretical 4.14%). Formula C_34_H_42_N_4_O_9_PtCl (881.2): *Anal.* Calc. C 46.34, H 4.80, N 6.36, Pt 22.14, Cl 4.02%, found C 46.66, H 4.72, N 6.69, Pt 22.26, Cl, 4.33%. IR (cm^−1^) 3319 ν(=NH); 2984, 2913, 2862 ν ^as,s^(CH_3_, CH_2_); 1620, 1350, ν ^as,s^(COO^−^). UV-Vis (8.10^−5^M in DMF) λ_max_ (log ε): 375 (4.04) 410 (4.89), 520 (3.83), 560 (3.74), 590 (3.70), 635 (3.57).

### 2.2. Analyses and physical measurements

C, H, N, and Cl^− ^ analyses were performed in the Elemental Analyses Laboratory in the University of Sofia. The Pt content was determined gravimetrically after treatment of the sample with concentrate H_2_SO_4_ and 30% H_2_O_2_. A pH-meter Radelkis OP-208 was used for the potentiometric measurements. The thermogravimetric measurements were performed on TGS-2 “Perkin Elmer” system and DSC was performed on 2C “Perkin Elmer” DS Calorimeter under argon. 

### 2.3. Spectroscopy and magnetic measurements

The absorption electronic, reflectance, and IR spectra (KBr-disks, 4000–400 cm^−1^, CsI-disk, 400–50 cm^−1^) were recorded on a UV-Vis “Carl-Zeiss, Jena,” Lambda 17 UV-VIS, and FTIR-Bruker IFS 113 V and “Perkin Elmer 983” spectrometers, respectively. The EPR spectra were obtained on an X-band “Bruker B-ER 420” spectrometer in the temperature range 100–298 K. Magnetic susceptibility was measured between 2 K and 300 K in magnetic field of 1 and/or 5 keys using commercial SQUID magnetometer (Quantum Design MPMS-XL) with sensitivity of 10^−7^ emu. The data were corrected for the diamagnetic response of the sample holder and for the diamagnetic contribution of the sample (Pascal's constants). X-ray photoemission spectra were recorded on an ESCALAB-MkII (VG Scientific) electron spectrometer ESCALAB-MkII (VG Scientific) with a base pressure of 1.10^−8^ Pa. C1s, O1s, N1s and Pt_4f _-photoemission lines were excited with an ${\text{MgK}}_{\alpha }$-radiation. All XPS spectra were calibrated using the C1s-core level at 285.0 eV as a reference.

### 2.4. Pharmacology

#### 2.4.1. Cell lines and culture conditions

In this study, the following human cell lines were used: SKW-3 (DSMZ no.: ACC 53)—T-cell leukemia—a derivative of KE-37 established from a patient with acute lymphoblastic leukemia; BV-173 (DSMZ no.: ACC 20)—chronic myeloid leukemia established from a CML patient in a lymphoblastic crisis; LAMA-84 (DSMZ no.: ACC 168)—chronic myeloid leukemia, originating from a CML patient in myeloid crisis. The cell lines were obtained from the German Collection of Microorganisms and Cell Cultures (DSMZ GmbH, Germany). The cells were grown as suspension-type cultures in controlled environment–RPMI-1640 medium, supplemented with 10% FBS and 2 mM L-glutamin, in cell culture flasks at 37°C with humidified atmosphere and 5% CO_2_. Cells were refereed with fresh medium two or three times a week in order to maintain logarithmic growth. 

#### 2.4.2. Cytotoxicity assessment, data processing, and statistics

The cell viability was assessed using the standard MTT (3-(4,5-dimethylthiazol-2-yl)-2,5-diphenyltetrazolium bromide) reduction assay as described by Mosmann [36] with minor modifications [37]. Stock solutions of *cis*-platin and the new platinum complexes were freshly prepared in DMSO and then diluted with corresponding growth medium. At the final dilutions, the solvent concentration never exceeded 1%.

#### 2.4.3. Apoptosis induction detection

Horizontal gel-electrophoresis of cytosolic DNA, isolated from SKW-3 cells treated with [Pt(Hp_−3H _)(H_2_O)_2_], **2** was performed in order to test the ability of the compounds under investigation to trigger programmed cell death (apoptosis). The procedure was carried out as described elsewhere [37].

The DNA fragmentation was monitored by a “Cell Death Detection” ELISA (Roche Diagnostics GmbH, Germany) as well. Cytosolic fractions of $1\times 10^{4}$ cells per group (treated or untreated) were used as antigen source in a sandwich ELISA, applying primary antihistone antibody-coated microplate and a secondary peroxidase-conjugated anti-DNA-antibody. The photometric immunoassay for histone-associated DNA fragments was executed according to the manufacturers instructions at 405 nm, using ELISA reader (Unican Titertec). The results are presented as the oligonucleosomal enrichment factor EF(%): $\text{E}\text{F}(\%)=({\text{A}}_{\text{T}\text{R}}/{\text{A}}_{\text{C}\text{O}})\times 100$, where A_TR_ is 405 nm absorption of treated samples; A_ CO_ is 405 nm absorption of control samples.

## 3. RESULTS AND DISCUSSION

### 3.1. Solution chemistry of the Pt(II)-hematoporphyrin interaction

**Figure 1:**
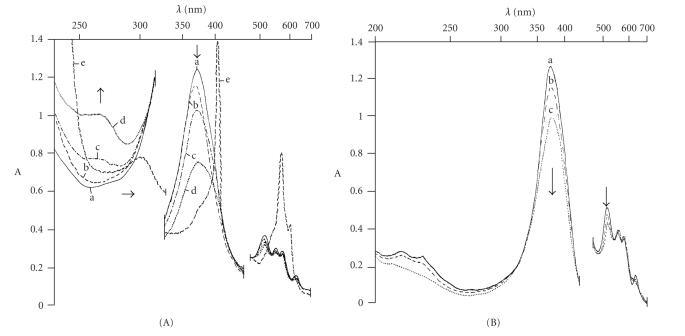
(A) Electronic absorption spectra of (a) aqueous alkaline solution of Hp; (b) 5 minutes after mixing of diammine(diaqua) platinum(II)hydroxide with the ligand (Pt:Hp = 2); (c) 2 days later; (d) 2 weeks later, at the end of the reaction; (e) after addition of 2M HNO3. (B) (a)–(c) Change of the electronic absorption spectra in the course the reaction K_2_PtCl_4_—hematoporphyrin (Pt:Hp = 1).

The Pt(II)-hematoporphyrin IX interaction was studied in aqueous-alkaline solution and aerobic conditions on light. The ligand for the syntheses was dissolved in $5\times 10^{-2}$ M KOH. Water solutions of *cis*-diammine(diaqua)platinum(II)hydroxide (obtained from *cis*-platin after precipitation of Cl^−^) and K_2_PtCl_4_ were used as initial Pt(II) complexes. The pH value of the reaction systems, obtained at different metal-to-ligand ratios was adjusted in the range 10.5–11.5 by addition of $5\times 10^{-2}$ M KOH solution. In all cases studied, the Pt(II)–hematoporphyrin interaction started with an increase of the acidity (ΔpH = 2–4). The changes in the electronic absorption spectra (Soret and Q-bands) during the interactions are depicted on Figures 1(A) and 1(B). 

The mixing of hematoporphyrin with diammine(diaqua)platinum(II)hydroxideat metal excess (Pt:Hp ≥ 2) is connected with a slight hypsochromic shift of the Soret band (369 nm) in comparison with the free ligand spectrum (374 nm) (Figure 1(A)a, b). Further spectral changes (Figure 1(A)c, d) in the course of the reaction followed the drop of the pH value. The Soret band (376 nm) decreased and broadened and a shoulder at ∼400 nm arose. A new band with growing intensity appeared at 270 nm during the interaction (Figure 1(A)c, d). At the end of the reaction, all absorption bands underwent a red shift and became less intensive and an additional decrease of pH value to ∼7.5 was established. A sequence of IV > III > II > I for the satellite Q-band intensity is observed. These spectral changes suggest that the porphyrin ring in the reaction product is distorted, and hence formation of sitting-atop- (SAT-) type complex with an asymmetrical coordination of the ligand through two adjacent pyrrole N atoms is most probably realized [25, 38]. 

**Figure 2:**
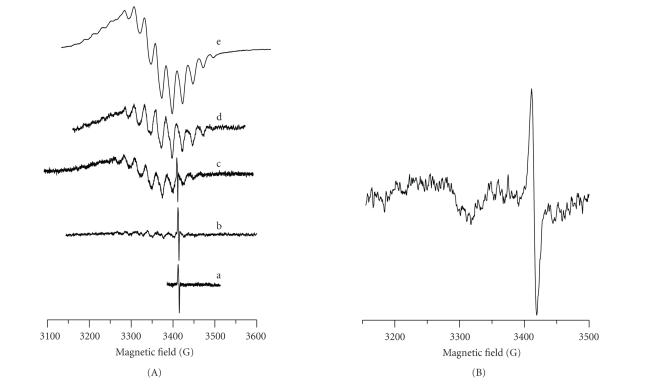
Frozen solution EPR spectra (at 130 K) of the reaction systems during the course of the interaction of (A) *cis*-diammine(diaqua)platinum(II)hydroxide—Hp (Pt:Hp ≥ 2), spectra taken after (a) three hours after mixing of the reagents; (b) nine hours; (c) a day; (d) one week; (e) two weeks, at the end of the reaction; (B) ${\text{PtCl}}_{4}^{-2}$-Hp (Pt:Hp = 1).

The reaction system was studied in a parallel way by EPR measurements. A narrow signal with parameters typical for a free radical (ΔH_ pp_ ∼ 3 G, with *g* = 2.009±0.001) was recorded in the EPR spectrum of a frozen sample taken one hour after the mixing of the reagents. The signal remains unchanged for a week in these conditions and indicates that a redox process takes also place in the system. Several hours after the start of the reaction together with the first signal, a new one appeared with increasing intensity (Figure 2(A)b, c). Nine superhyperfine lines due to the interaction of the unpaired electron of Pt(III) with four ^14^N-nuclei (I = 1) were readily observed in the perpendicular region (Figure 2). The signal most probably corresponds to a rhombic symmetry as follows from the slight splitting observed in the perpendicular region (Figure 2(A)d). A week later, an intensive signal with parameters closed to the former one could be only found in the spectrum (Figure 2(A)e). This signal corresponds to an axial symmetry of the complex with g_∥_ > *g*_⊥_ > 2.0023 and *g* ${\left({\text{d}}_{{\text{z}}^{2}}\right)}^{1}$ ground state. Nine superhyperfine lines from four ^14^N-nuclei (I = 1, A_⊥_(N) = 21.0 × 10^−4^ cm^−1^) were observed in the perpendicular region. The superhyperfine structure in the parallel region due to ^14^N (A_∥_(N) = 24.9 × 10^−4^ cm^−1^) overlaps the hyperfine structure due to ^195^Pt(I = 1/2, A_∥_ ∼ 50 × 10^−4^ cm^−1^). The hyperfine structure (A(Pt)) was better resolved in the parallel region than in the perpendicular one (A_⊥_(Pt) ∼ 60 × 10^−4^ cm^−1^). Hence, a stable Pt(III) complex is the product of the *cis*-diammine(diaqua)platinum(II)hydroxide interaction with hematoporphyrin in aqueous alkaline medium, in which complex platinum(III) is surrounded by four N donor atoms (Figure 2(A)e). The axial signal symmetry is in accordance with the symmetrical coordination of the ligand to Pt(III) via the four pyrrole N-atoms in the porphyrin macrocycle.

An intermediate Pt(III) complex with a lower symmetry signal was recorded in the course of this reaction. In this complex, platinum(III) is coordinated again to four nitrogen atoms. The lower symmetry is most probably due to a SAT complex formation where two of the nitrogens come from adjacent porphyrin pyrroles and the other two from the ammine groups of the initial Pt(II) complex. 

The interaction of ${\text{PtCl}}_{4}^{-2}$ with Hp in aqueous alkaline solution was studied at equimolar ratio of the reagents. It was found that the acidity of the reaction mixture increases faster than in the case when diammine(diaqua)platinum(II)hydroxide was used. During the reaction course, the intensity of the characteristic absorption bands decreased (Figure 1(B)a–c). A narrow EPR signal (ΔH_pp_ ∼ 8 G) with *g* = 1.986 was recorded in the EPR spectra of the frozen reaction system (130 K) again due to a stable free radical (Figure 2(B)). In addition, a second broad (ΔH_pp_ ∼ 60 G) and low intensive signal appeared in a magnetic field about 3280 G (*g* ∼ 2.06) several hours later most probably due to a Pt(III)-complex formation. In the spectrum, no superhyperfine splitting from ^14^N was observed, and hence the ligand in this complex is coordinated through the carboxylic acid groups outside the porphyrin macrocycle.

### 3.2. Structural characterization of the complexes

Three different Pt(III)-hematoporphyrin complexes have been isolated during the interactions discussed above. The neutral complex *cis*-[Pt(NH_3_)_2_(Hp_−3H_)(H_2_O)_2_]·H_2_O **1** was precipitated from the reaction mixture of *cis*-diammine(diaqua)platinum(II) hydroxide and hematoporphyrin (Pt:Hp = 4) after a spontaneous decrease of pH down to 8. The complex [Pt(Hp_−3H _)(H_2_O)_2_]·H_2_O **2** is the main product from the reaction mixture at Pt:Hp = 2 molar ratio. The complex [Pt((O,O)Hp_−2H_)Cl(H_2_O)_3_] **3** was obtained from the reaction system ${\text{PtCl}}_{4}^{-2}$-Hp taken in equimolar ratio. The complexes **2** and **3** were isolated as powders after addition of HNO_3_ and HClO_4_. The contents of water and NH_3_ for complex **1** and of Cl^−^ for complex **3** were determined by thermogravimetric and calorimetric measurements (Table 1). The relatively high temperatures of dehydration (above 130°C) as well as the thermal effects indicated that some water molecules are bound in the inner coordination sphere. The processes of the removal of water and ammonia molecules are endothermic, while that for Cl^−^ as HCl is an exothermic one.

The bands in the UV-V is absorption and reflectance spectra of the complexes obtained correspond to those from the electronic spectra recorded in the solution during the interactions (Figure 1). The three Pt(III)-Hp complexes have shown the characteristic Soret band about 400 nm with a molar absorptivity up to $1\times 10^{5}$ mol^−1^.L.cm^−1^. In the spectrum of the complex **1**, the Soret band is broadened. Four Q-bands could readily be distinguished in the range of 500–650 nm. The intensity of the bands IV < III ∼ II > I differs from the etio-type free ligand spectrum and all bands are red-shifted in comparison with the free ligand spectrum. The presence of all four bands indicates a relatively low symmetry of the porphyrin ring, due to an unsymmetrical coordination of the hematoporphyrin ligand to platinum as it could be expected for SAT-type complexes [25, 38]. The isolation of the complex **2** by adding of acid resulted in a reduction of the number of the Q-bands to two (Figure 1(A)e). A decrease of the Q-bands number was observed in the electronic and reflectance spectra of the isolated complex. This fact could be explained with increasing of the symmetry of the porphyrin ring through coordination of platinum to the four pyrrole nitrogen atoms. The complex **3** showed absorption spectrum of an etio type similar to that of the free ligand with a sequence of the Q-bands intensity of IV > III > II > I. In addition, a shoulder at 375 nm also appeared. The spectrum obtained indicates that platinum is most probably coordinated to the peripheral carboxylic groups of the hematoporphyrin ring.

**Figure 3:**
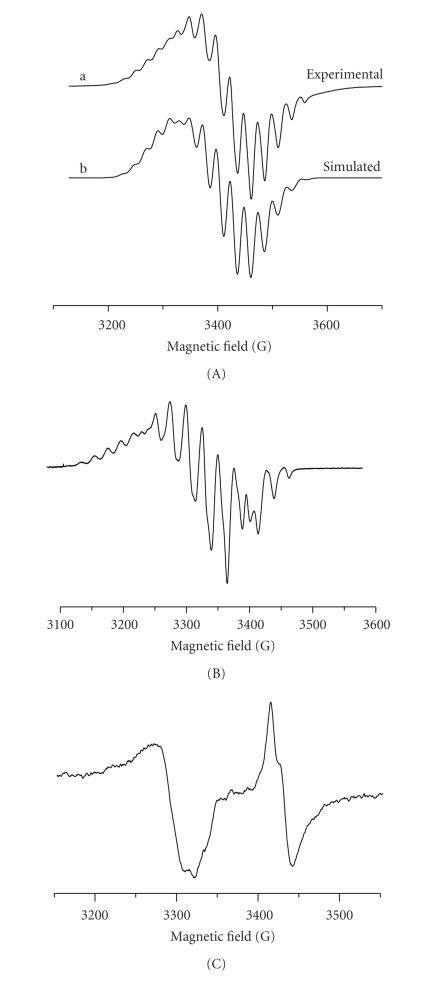
EPR spectra of polycrystalline samples of the complexes: (A) [Pt(III)(Hp_−3H _)(H_2_O)_2_]·H_2_O **2** (298 K) : (a) experimental; (b) simulated; (B) *cis*-[Pt(III)(NH_3_)_2_(Hp_−3H _)(H_2_O)_2_]·H_2_O **1** (130 K); (C) [Pt((O,O)Hp_−2H_)Cl(H_2_O)_3_] **3** (110 K).

The powder EPR spectra of the complexes are shown on Figure 3. The parameters determined from the experimental spectra and those used in the simulation procedure are shown in Table 2. The complex **2** shows (Figure 3(A)a) an anisotropic signal ($g_{∥}=2.113$ and $g_{⊥}=2.038$) with temperature-dependent intensity, which is due to Pt(III). The signal possesses an axial symmetry with $g_{∥}>g_{⊥}>2.003$ and ${\left({\text{d}}_{{\text{z}}^{2}}\right)}^{1}$ ground state. Nine superhyperfine lines due to interaction of uncoupled electron with four ^14^N (I = 1) were observed both in perpendicular and parallel regions. The hyperfine signal structure due to ^195^Pt (I = 1/2) overlaps the superhyperfine structure and is better resolved in the parallel region. Simulated EPR spectrum (Figure 3(A)b) was obtained using the parameters of the experimental EPR spectrum (Figure 3(A)a). The model is based on the assumption that platinum is in oxidation state +3 (I = 1/2), with natural abundance of ^195^Pt 33.8%. Platinum(III) is assumed to be coordinated via four nitrogens of the porphyrin macrocycle [39]. Nitrogen nuclei (^14^N, I = 1) are present in two groups of magnetically equivalent nuclei, each group containing two opposite nitrogen nuclei from the porphyrin cycle. The simulation procedure was performed by variation of the principal values of *g*-tensor, nuclear hyperfine tensors (A(Pt)), and nuclear superhyperfine tensors (A(N)). The best fit was achieved with the parameters shown in Table 2.

The EPR signal of complex **1** is anisotropic but with a lower symmetry and an additional splitting in the perpendicular region. Nine superhyperfine lines due to the interaction with four ^14^N nuclei were observed. In the case of rhombic symmetry and presence of superhyperfine coupling with four ^14^N nuclei, the number of lines increases and the intensity of the superhyperfine lines from ^195^Pt nuclei (33.8%) decreases. Hence, the principal values of nuclear hyperfine tensor (^195^Pt) could not be determined from the experimental spectrum. The analysis of the signal was based on the assumption for a rhombic symmetry. The values of the rhombic *g*-tensor and rhombic nuclear superhyperfine tensor A(N) were determined from the experimental spectrum (Table 2). The decrease of the symmetry is most probably due to the coordination of unequivalent N atoms in the Pt equatorial plane of platinum.

The EPR spectrum of complex **3** consists of two signals. The lowfield signal corresponds to Pt(III) complex with a rhombic symmetry and *g*-values given in Table 2. The EPR linewidth is close to the hyperfine splitting constant, and for this reason the hyperfine structure from ^195^Pt is not resolved. The absence of a superhyperfine structure from ^14^N nuclei could be related to the fact that Pt(III) is coordinated outside the porphyrin ring. The highfield signal corresponds to a free radical with an axial symmetry and parameters $g_{∥}=2.000$ and *g*_⊥_ = 1.980.

**Figure 4:**
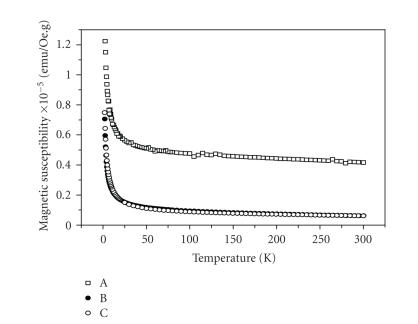
Plots of the molar magnetic susceptibility (χ) versus temperature for the complexes: (A) *cis*-[Pt(III)(NH_3_)_2_(Hp_−3H _)(H_2_O)_2_]·H_2_O **1**; (B) [Pt(III)(Hp_−3H_)(H_2_O)_2_]·H_2_O **2**; (C)[Pt((O,O)Hp_−2H _)Cl(H_2_O)_3_] **3**.

Magnetic susceptibility (χ) decreases monotonically with increasing temperature (Figure 4), thus suggesting paramagnetic behavior in the range of 2–300 K and octahedral structure for all complexes studied. The effective magnetic moments, $\mu_{\text{eff}}$, were estimated taking into account the diamagnetic corrections via the tabulated Pascal constants ($4.08\times 10^{-4}$ emu/mol; $-3.72\times 10^{-4}$ emu/mol; and $-3.98\times 10^{-4}$ emu/mol for complexes **1**, **2**, and **3**, resp.). The calculated values of $\mu_{\text{eff}}=2.19\;\mu_{\text{B}}$, $1.54\;\mu_{\text{B}}$ and $1.73\;\mu_{\text{B}}$, for the complexes **1**, **2**, and **3**, respectively, are in an agreement with the spin-only value $\mu_{\text{so}}=1.73$.

Selected data from the X-ray photoemission spectra of the free ligand and the complexes studied are present in Table 3. The two peaks for the N 1s binding energy at 400.0 and 398 eV in the free ligand spectrum are usually assigned to the pyrrole (H−N<) and the aza (−N=) nitrogens. The higher energy peak belongs to the pyrrole nitrogens because of their higher electronegativity [24].

The presence of only one N 1s peak in complex **2** indicates that all nitrogens in this compound are equivalent. The intermediate value of the N 1s binding energy (399.0 eV) is due to coordination of the four N atoms to platinum and formation of metalloporphyrin-type complex by incorporation of platinum in the porphyrin ring.

**Scheme 1:**
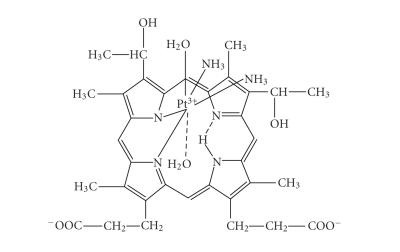


Three different peaks could be distinguished in the N 1s spectrum of complex **1**. The two lower energy peaks at 399.2 eV and 398.4 eV were assigned to two pairs of equivalent hematoporphyrin nitrogens. The N 1s binding energy peak at 399.2 is assigned to nitrogens coordinated to platinum. The lowest energy peak that is close to that of the free ligand aza nitrogens is assigned to uncoordinated hematoporphyrin nitrogens. The equivalence of these two nitrogens as well as the decrease of their electronegativity in comparison with the free pyrrole nitrogens could be explained with delocalization of electron density by H-bonding formation (Scheme 1). The highest binding energy peak at 400.5 eV shows the presence of nitrogens with a higher electronegativity with respect of the free ligand nitrogens and was assigned to coordinated ammine nitrogens. 

**Scheme 2:**
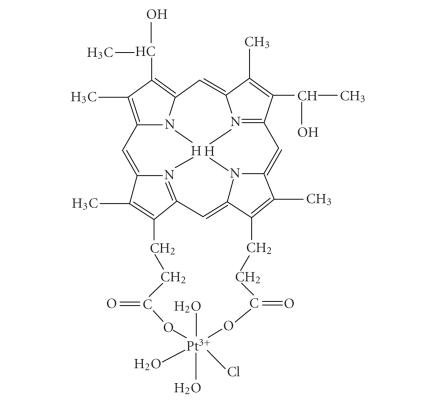


The coordination of the ligand by peripheral carboxylic groups in complex **3** was proved by the presence of low energy peak in the O 1s spectrum at 529.0 eV. The peaks in the interval of 532.2–532.6 eV for the free ligand and the complexes O 1s spectra are due to uncoordinated ligand oxygens and the presence of water molecules. The peak at 400.3 eV in the N 1s spectrum of complex **3** is assigned to the four equivalent uncoordinated hematoporphyrin nitrogens. The relatively high N 1s binding energy and the equivalence of the four nitrogens could be considered as a result of a significant metal to ligand electron-density transfer and delocalization by H-bonding formation (Scheme 2).

The Pt 4f spectra of the complexes are resolved into spin-orbit pairs with splitting of 3.3 eV for all complexes studied. The determined Pt 4f_7/2_ binding energies values for the three complexes in the range of 73.1–73.8 eV are in accordance with the formation of the +3 oxidation state. 

The IR data (Table 4) have shown identical ν(OH) and ν ^as,s^(CH_3_,CH_2_) stretching vibrations for the hydroxyethyl, methyl, and propionic side chains as well as in-plane and out-of-plane porphyrin skeletal vibrations. The coordination of H_2_O in the complexes follows from the presence of ν ^as,s^(H_2_O) in the range of 3350–3450 cm^−1^and of δ(H_2_O) at 1600–1630 cm^−1^. The assignments of the bands are made in accordance with the IR data and NCA published for metalloporphyrins [40].

The ν(NH) band at 3312 cm^−1^ disappears in the spectrum of **2**, and thus indicates a metal insertion into the porphyrin macrocycle and the formation of the metalloporphyrin-type complex. Bands of carboxylate ion (ν ^as,s^(COO^−^) 1563, 1352) and carbonyl stretch (1704 cm^−1^) from the protonated COOH are present in the same spectrum. This fact shows that both protonated and deprotonated carboxylic groups participate in the complex. The coordination via pyrrole N-atoms is supported also by the presence of a strong absorption band at 441 cm^−1^ assigned to Pt−N stretching vibrations. The bands in the far-infrared spectrum in the range 390–230 cm^−1^ were assigned to stretching Pt−O and deformation mode vibrations of the coordinated H_2_O molecules [40].

Conversely, in the IR spectrum of **3**, the presence of ν(NH) at 3319 cm^−1^ shows coordination outside the porphyrin macrocycle. In addition, a shift of the C=O porphyrin-carboxylic acid bands at 1620 and 1350 cm^−1^ indicates coordination through side chain deprotonated carboxylic groups. A value of 270 cm^−1^ for Δ = (ν ^as^(COO^−^)−ν ^s^(COO^−^) proves unidentate coordination [40] of the carboxylic groups. The bands at 366 and 324 cm^−1^ as well as those at 258 and 210 cm^−1^ are assigned to stretching vibrations of Pt−O' (O' belongs to coordinated carboxylic groups) and Pt−Cl. Other far-infrared bands at 483, 461, 453, 388, and 231 could be assigned to stretching Pt−O and deformation mode vibrations of the coordinated in-plane and in axial positions H_2_O-molecules [40].

The IR spectrum of **1** shows a characteristic ν(NH) absorption band of uncoordinated pyrrole at 3303 cm^−1^. The C=O absorption is shifted to 1552 and 1407 cm^−1^ for ν ^as,s^(COO^−^) stretching vibrations. The value of Δ = (ν ^as^(COO^−^)−ν ^s^(COO^−^) = 145 cm^−1^corresponds to the presence of deprotonated uncoordinated carboxylic groups. Besides, the bands due to coordination of NH_3_ molecules were observed in the range 3235–3090 cm^−1^ and at 1605 cm^−1^. These spectral data correspond to a SAT complex formation, where platinum is coordinated partially to some of the porphyrin nitrogens, part of them being still protonated. The Pt(III) coordination sphere includes also two NH_3_-molecules from the initial Pt(II)-complex in a *cis*-position as follows from the presence of two pairs of antisymmetric and symmetric stretching vibrations of Pt−N_2_ and Pt−N'_2_(N' belongs to NH_3_) at 444, 425 cm^−1^ and 394, 364 cm^−1^ [40]. The other bands in the range 316–160 cm^−1^could be assigned to stretching Pt−O and deformation mode vibrations of the coordinated H_2_O molecules.

Summarizing all experimental data obtained, it can be concluded that the interaction between Pt(II) and Hp in aqueous alkaline solution proceeds in a different way depending mainly on the type of the initial Pt(II) species and the metal-to-ligand ratio. The complexation process is accompanied by a parallel redox process leading to formation of Pt(III) species and organic radicals, together with a considerable decrease of pH. The final products of the overall Pt(II)-Hp interaction are three Hp-complexes of Pt(III).

The first SAT-type complex **1** precipitatesin alkaline medium after spontaneous decrease of initial pH from 11 to ∼8, M:L molar ratio of 4, platinum being introduced as *cis*-diammine(diaqua)platinum(II) hydroxide. In these conditions, the protons of the carboxylate groups dissociate and the ligand reacts as twofold deprotonated species [20]. Its coordination to Pt(III) is realized through two adjacent porphyrin pyrrole nitrogens, substituting one pyrrole' hydrogen. The PtN_4_-unit in the coordination sphere is formed with participation also of two NH_3_-molecules in a *cis*-position (Scheme 1).

**Scheme 3:**
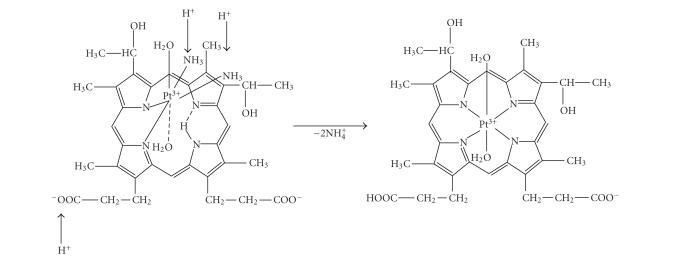


The metalloporphyrin-type complex **2**—the main product from the same reaction mixture but at Pt:Hp = 2 molar ratio—was isolated in a solid state using HNO_3_ (Scheme 3). In acidic medium, the coordinated NH_3_ molecules leave the inner coordination sphere as NH_4_^+^ and Pt(III) coordinate to the other two pyrrole nitrogen atoms by substitution of a second proton. The complex was precipitated through protonation of one of the side carboxylate groups.

The complex **3** was obtained by the reaction of ${\text{PtCl}}_{4}^{-2}$ and hematoporphyrin in equimolar ratio. Because of the faster base hydrolysis of ${\text{PtCl}}_{4}^{-2}$ and the Pt(III) preference for O-donors (in comparison with Pt(II)), the coordination here is realized via deprotonated carboxylic groups out of the porphyrin macrocycle. The equatorial coordination plane of platinum includes also Cl^−^ and H_2_O molecules (Scheme 2).

A distorted octahedral structure with H_2_O molecules disposed in axial position is suggested for all three complexes.

### 3.3. Cytotoxic activity of the complexes

The experimental data from the cytotoxicity investigation were fitted to sigmoidal dose-response curves. The correspondingly calculated IC_50_ values are summarized in Table 5. The novel platinum(III) complexes under investigation exerted cytotoxic effects against the panel of leukaemic cell lines in a concentration-dependent manner. Against both BV-173 and LAMA-84 cells, the compounds **1** and **2** displayed significant cytotoxic efficacy with IC_50_ values comparable to those of the referent cytotoxic agent *cis*-DDP. Furthermore, the maximal efficacy of **1** and **2** , estimated at 50 *μ*M, was superior to that of *cis*-platin against both LAMA-84 and BV-173. It is noteworthy that despite the different cell types of LAMA-84 (myeloid) and BV-173 (lymphoid), these lines share the same origin, being both isolated from chronic myeloid leukemia (CML) patients in blast crisis. Conversely, both cell lines are characterized via the expression of the characteristic for CML BCR-ABL protein, a nonreceptor tyrosine kinase whose constitutive activation renders the cells less responsive to proapoptotic stimuli, including chemotherapy agents [41, 42]. The cytotoxicity of *cis*-platin, being circa twofold less active on LAMA-84 than in BV-173 reflects the well-known discrepancies of the degree of BCR-ABL expression, being more pronounced in the former cell line [41]. In a dissimilar fashion, both **1** and **2** share practically identical potency in both cell lines, which clearly indicates that the level of BCR-ABL expression does not affect their cytotoxicity significantly.

The complex **3** was found to be far less active on molar basis against LAMA-84 and BV-173 This complex causes 50% reduction of cell viability at 2-3 times higher concentrations as compared to **1** and **2**. The SKW-3 cell line demonstrated higher sensitivity to the complex **3**, with an IC_50_ value being twice that of *cis*-DDP, whereas **1** and **2** induced half-maximal effects at 2-fold higher concentrations.

**Figure 5:**
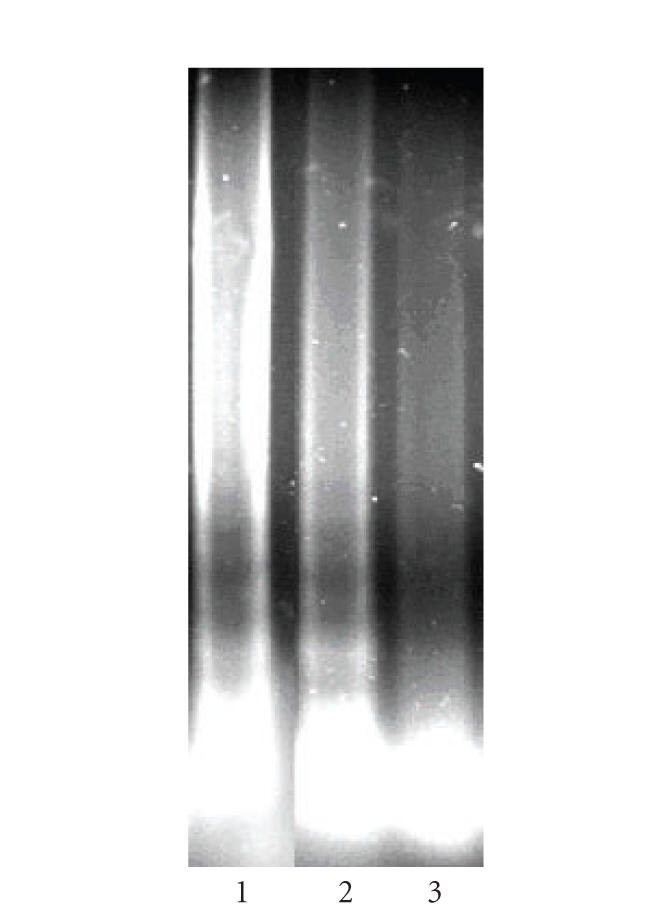
DNA-laddering, a hallmark feature of apoptosis, following exposure of SKW-3 cells to complex **2**. DNA was extracted from the cytosolic fraction of SKW-3 cells following 24-hour treatment of complex **2**, at a concentration of 10 *μ*M (lane 1) and 5 *μ*M (lane 2), versus control (0.5% DMSO treated, lane 3) and analyzed via 0.8% agarose gel electrophoresis, ethidium bromide staining, and UV-transillumination.

The results from the apoptosis assay are depicted on Figure 5. The detected DNA-laddering showed that the observed cytotoxicity of the complex **2** is at least partly mediated via the recruitment of the apoptotic cell signaling pathways in lymphoid SKW-3 cells.

**Figure 6:**
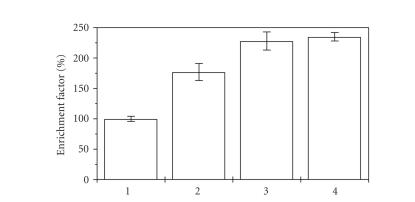
Enrichment of SKW-3 cytosole with histone-associated mono- and oligonucleosomal DNA-fragments after 24-hour treatment with complex **2** at concentration 12.5 *μ*M (**2**), 25 *μ*M (**3**), or 50 *μ*M (**4**), versus the untreated control (**1**). Each column represents the arithmetic mean ± standard deviations of 3 separate experiments.

In order to elucidate the proapoptotic activity of **2** in a more quantitative manner, its ability to induce oligonucleosomal DNA fragmentation was analyzed by means of “Cell Death Detection” ELISA (Roche Diagnostics). The obtained results have shown that a 24-hour treatment of SKW-3 cells with **2** (at 12.5, 25, or 50 *μ*M) leads to a significant enrichment of the cytosole with histone-associated mono- and oligonucleosomal DNA-fragments (Figure 6). These findings together with the established DNA-laddering unambiguously indicate that the recruitment of the programmed cell death signaling pathways plays a pivotal role for the cytotoxic mode of action of the tested complex compound.

## Figures and Tables

**Table 1:** Thermogravimetric and calorimetric data for the complexes **1**, **2**, and **3**.

Complex	*Processes of decomposition	Temp. interval (°C)	E (kcal·mol^−1^)	Thermal effects
*cis*-[Pt(NH_3_)_2_(Hp_−3H_)(H_2_O)_2_]·H_2_O, **1**	I	95–130	23.3 for 3H_2_O	Endo
	II	130–260		Endo
	III	260–295	53.4 for 2NH_3_	Endo
[Pt(Hp_−3H_)(H_2_O)_2_]·H_2_O, **2**	IV	100–130	35.9 for 3H_2_O	Endo
	V	130–250		Endo
[Pt((O,O)Hp_−2H_)Cl(H_2_O)_3_],**3**	VI	100–161	76.0 for 2H_2_O	Endo
	VII	161–225	19.3 for 1HCl	Exo
	VIII	225–250	9.3 for 1H_2_O	Endo

*Processes of thermal decomposition:

^(I)^ *cis*-[Pt(NH_3_)_2_(Hp_−3H _)(H_2_O)_2_]·H_2_O → *cis*-[Pt(NH_3_)_2_(Hp_−3H _)(H_2_O)_2_] + H_2_O

^(II)^ *cis*-[Pt(NH_3_)_2_(Hp_−3H _)(H_2_O)_2_] → *cis*-Pt(NH_3_)_2_(Hp_−3H_) + 2H_2_O

^(III)^ *cis*-Pt(NH_3_)_2_(Hp_−3H _) → Pt(Hp_−3H_) + 2NH_3_

^(IV)^ [Pt(Hp_−3H_)(H_2_O)_2_]·H_2_O → [Pt(Hp_−3H_)(H_2_O)_2_] + H_2_O

^(V)^ [Pt(Hp_−3H_)(H_2_O)_2_] → Pt(Hp_−3H_) + 2(H_2_O)

^(VI)^ [Pt((O,O)Hp_−2H _)Cl(H_2_O)_3_] → [Pt((O,O)Hp_−2H _)Cl(H_2_O)] + 2H_2_O

^(VII)^ [Pt((O,O)Hp_−2H_)Cl(H_2_O)] → [Pt((O,O)Hp_−3H_)(H_2_O)] + HCl

^(VIII)^ [Pt((O,O)Hp_−3H_)(H_2_O)] → Pt((O,O)Hp_−3H_) + H_2_O

**Table 2:** EPR parameters from the experimental spectra and parameters used in the simulation procedure.

Complexes					
*cis*-[Pt(III)(NH_3_)_2_(Hp_−3H_)(H_2_O)_2_]·H_2_O **1**					
*g*_1_	*g*_2_	*g*_3_	${\text{A}}_{1}(\text{N})\times 10^{-4}$ cm^−1^	${\text{A}}_{2}(\text{N})\times 10^{-4}$ cm^−1^	${\text{A}}_{3}(\text{N})\times 10^{-4}$ cm^−1^
2.126	2.024	2.019	20.1	22.7	23.1

[Pt(III)(Hp_−3H _)(H_2_O)_2_]·H_2_O **2**					
*g*_∥_	*g*_⊥_	${\text{A}}_{∥}(\text{Pt})\times 10^{-4}$ cm^−1^	${\text{A}}_{⊥}(\text{Pt})\times 10^{-4}$ cm^−1^	${\text{A}}_{∥}(\text{N})\times 10^{-4}$ cm^−1^	${\text{A}}_{⊥}(\text{N})\times 10^{-4}$ cm^−1^
2.113±0.001	2.038±0.001	47.92±0.03	55.41±0.03	14.80±0.03	14.90±0.03

[Pt((O,O)Hp_−2H _)Cl(H_2_O)_3_] **3**					
*g*_1_	*g*_2_	*g*_3_	$g_{∥}$ (radical)	*g*_⊥_ (radical)	
2.111	2.065	2.046	2.000	1.980	

**Table 3:** Selected data from X-ray photoemission spectroscopy.

Compounds	Pt 4f_7/2_ [eV]	N 1s [eV]	Assignment	O 1s [eV]
Hematoporphyrin IX		400	>N−H	532.2
		398	=N−	
*cis*-[Pt(III)(NH_3_)_2_(Hp_−3H _)(H_2_O)_2_]·H_2_O **1**	73.2	400.5	NH_3_	532.6
		399.2	>N−Pt	
		398.4	>N$\text{H}\cdots \underset{|}{\text{N}}=$	
[Pt(III)(Hp_−3H _)(H_2_O)_2_]·H_2_O **2**	73.1	399.0	>N−Pt	532.6
[Pt((O,O)Hp_−2H _)Cl(H_2_O)_3_] **3**	73.8	400.3	>N$\text{H}\cdots \cdots \cdots \underset{|}{\text{N}}=$	532.5
				529.0

**Table 4:** Selected frequencies from the infrared spectra of the free ligand and Pt(III)—hematoporphyrin IX complexes (cm^−1^).

Hp	Complex **1**	Complex **2**	Complex **3**	Assignment
3620	3444	3445	3390	ν(OH) + ν ^as,s^(H_2_O)
3432				

3312	3303	—	3319	ν(NH)

—	3235	—	—	ν ^as,s^(NH_3_)^1^
	3218			
	3172			
	3093			

2969	2964	2968	2984	ν ^as,s ^(CH_3_,CH_2_)
2920	2924	2915	2913	
2861	2855	2875	2862	
2832sh		2820sh		

1715	—	1704		ν(−C=O)
—	1552	1563	1620 ν(−C=O) free	ν ^as,s^(COO^−^)
	1407	1352	1350 ν(−C−O) coord.	
	∼1605 br	∼1630 br	∼1600 br	δ(NH_3_)^1^ + δ(H_2_O)

Far-IR region	ν(MN) + ν(MN') + ν(MO)	ν(MN) + ν(MO)		ν(MN′) + ν(MO) + ν(Pt−Cl)
	444st	441st		483
	425			461
		391		452
	394	366		
	364			388
		318		366st
	316	294		324st
	293			
	269	268		258
		239		231w
	234			210
	214	187		
		165		
	186			
	160			
	121			
	106			

^1^Complex 1

**Table 5:** Comparative cytotoxic activity of the investigated platinum (III) complexes **1**, **2**, and **3** versus *cis*-DDP in a panel of human tumour cell lines after 72 hours (MTT-dye reduction assay).

Compound	IC_50_ value (μM)^a^		
	SKW-3	LAMA-84	BV-173
*cis*-DDP	10.21±3.41	17.42±1.38	10.47±4.6
**1**	39.81±2.17	16.46±1.94	15.96±2.05
**2**	37.78±1.15	14.68±2.89	15.56±0.72
**3**	21.72±2.66	58.55±6.41	32.82±7.01

^a^Arithmetic mean ± standard deviation of 6 independent experiments.
